# Hamstring injuries

**DOI:** 10.1093/jhps/hnv026

**Published:** 2015-06-06

**Authors:** Carlos A. Guanche

**Affiliations:** Southern California Orthopedic Institute, CA, USA

## Abstract

There is a continuum of hamstring injuries that can range from musculotendinous strains to avulsion injuries. Although the proximal hamstring complex has a strong bony attachment on the ischial tuberosity, hamstring injuries are common in athletic population and can affect all levels of athletes. Nonoperative treatment is mostly recommended in the setting of low-grade partial tears and insertional tendinosis. However, failure of nonoperative treatment of partial tears may benefit from surgical debridement and repair. The technique presented on this article allows for the endoscopic management of proximal hamstring tears and chronic ischial bursitis, which until now has been managed exclusively with much larger open approaches. The procedure allows for complete exposure of the posterior aspect of the hip in a safe, minimally invasive fashion.

## INTRODUCTION

Hamstring injuries are common in athletic populations and can affect all levels of athletes [1, 2, 3–7]. There is a continuum of hamstring injuries that can range from musculotendinous strains to avulsion injuries [3, 4]. By definition a strain is a partial or complete disruption of the musculotendinous unit [4]. A complete tear or avulsion, in contrast, is a discontinuity of the tendon bone unit. Most of hamstring strains do not require surgical intervention and resolve with a combination of modalities and rest [3–7].

With the exception of the short head of the biceps femoris, the hamstring complex originates from the ischial tuberosity and inserts distally below the knee on the proximal tibia. The tibial branch of the sciatic nerve innervates the semitendinosus, semimembranosus and the long head of biceps femoris, while the short head of the biceps femoris is innervated by the peroneal branch of the sciatic nerve [5].

**Fig. 1.**
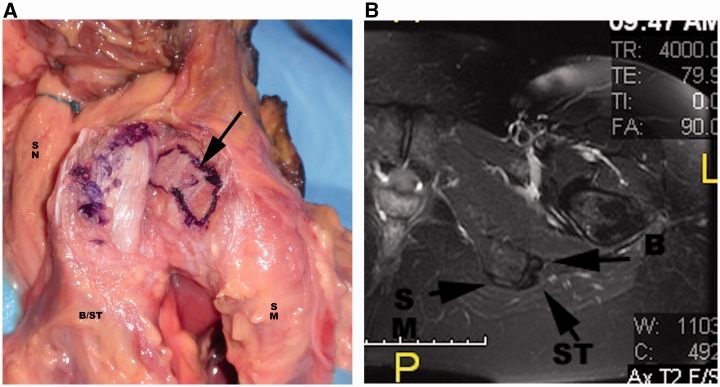
(A) Cadaveric dissection of the ischium in a left hip, viewed from the posterior aspect. The arrow points to the origin of the Biceps/Semitendinosus (B/ST), which has been elevated and retracted laterally. (B) Axial T2 weighted view depicting the anatomy of the hamstring origin in a left hip. SN: Sciatic Nerve; SM (origin of Semimembranosus); B: Biceps origin.

The proximal hamstring complex has a strong bony attachment on the ischial tuberosity (Fig. 1). The footprint on the ischium includes the semitendinosus and the long head of biceps femoris beginning as a common proximal tendon and footprint, while there is a distinct semimembranosus footprint [8]. The semimembranosus footprint is lateral (and anterior) to the crescent-shaped footprint of the common insertion of the semitendinosus and long head of the biceps femoris (Fig. 1).

The history of an acute injury usually involves a traumatic event with forced hip flexion and knee in extension, as is classically observed in waterskiing, but can occur with other activities [9–12].

Whereas acute avulsions are clinically obvious, the degenerative tears of the hamstring origin are more insidious in onset and are commonly seen as an overuse injury. The mechanism of injury is presumably repetitive irritation of the medial aspect of the hamstring tendon (typically along the lateral aspect of the tuberosity, where the bursa resides) ultimately causing an attritional tear of the tendon [11].

Commonly, athletes with proximal hamstring tendon tears describe a popping or tearing sensation with associated acute pain and bruising over the posterior hip [13, 14]. They may also complain of a pins and needles sensation in the sciatic nerve distribution, much like sciatica [14, 15]. This may be due to the acute compression of a hematoma in the proximity of the sciatic nerve or chronic scarring and tethering of the tendon to the nerve. Similarly, symptoms of ischial bursitis include buttock pain or hip pain, and localized tenderness overlying the ischial tuberosity with occasional sciatica symptoms.

**Fig. 2.**
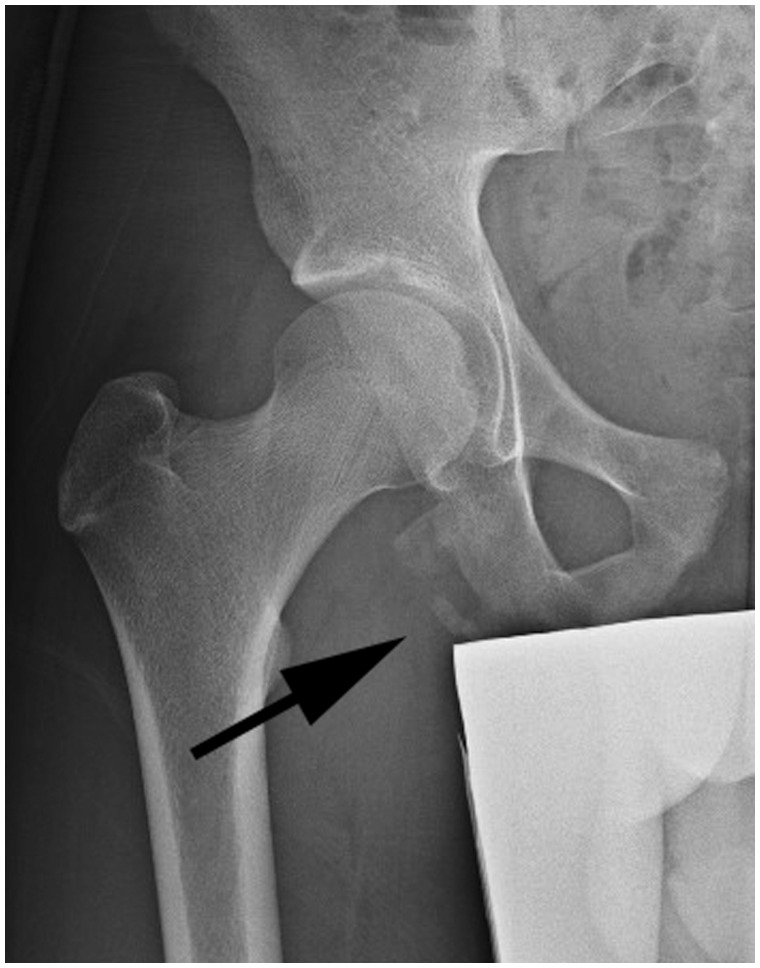
AP of pelvis showing bilateral bony avulsions of the ischial tuberosities in a 14-year-old athlete.

Once the diagnosis is suspected, standard radiographs of the pelvis and a lateral of the affected hip are performed to rule out any apophyseal avulsions, particularly to the ischial tuberosity in younger patients (Fig. 2). Most commonly, no fractures are identified and MRI is used to assess the proximal hamstring insertion on the ischial tuberosity, where several types of injuries may be seen. A complete rupture of all three tendons is common and is easily identified on MRI.

**Fig. 3.**
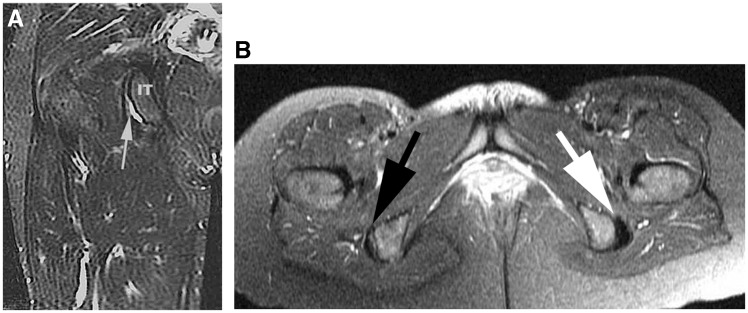
MRI views of a partial insertional tear with a sickle sign, indicating fluid in the ischial bursa. (A) A coronal view, T2-weighted view of a right hip showing the sickle sign (white arrow). IT: Ischial tuberosity. (B) Axial view, T2 weighted, showing both ischial tuberosities. Note the right side (black arrow) showing the sickle sign and the normal left side (white arrow).

Partial hamstring origin tears, however, are more difficult to delineate. This is particularly the case in two tendon tears, which commonly have an associated musculotendinous junction injury to the third tendon. The most common situation is an avulsion of the common semitendinosus and biceps origin, with the semimembranosus remaining intact [7]. In addition, partial insertional tears without any significant retraction can be seen on MRI as a sickle sign. These are typically partial avulsions of the semimembranosus (Fig. 3).

Nonoperative treatment of proximal hamstring injuries is most commonly recommended in the setting of low-grade partial tears and insertional tendinosis. In general, non-operative treatment in higher level athletes has not been effective, as up to 80% complain of ongoing cramping and weakness [16]. Initial treatment consists of active rest, oral non-steroidal anti-inflammatory medications and a physical therapy program [17]. If the patient is unable to progress with this program, an ultrasound-guided corticosteroid injection may be used and has been shown to provide initial relief in up to 50% of patients at 1 month [18]. Failure of non-operative treatment of partial tears may benefit from surgical debridement and repair [16].

There are several series and descriptions of open surgical techniques that are available in the literature [12–14, 19–21]. To date, there has been no report of endoscopic management of these injuries. The endoscopic approach has been developed over the last 18 months, where the procedure has been used in a group of 15 patients. All patients underwent the surgery as described with no need to abandon the procedure as a result of failure of visualization of any of the structures. All patients underwent suture anchor fixation with no anchor complications to date. There were two patients that initially complained of numbness over the posterior thigh with resolution of their symptoms by 6 weeks post-operatively. There were no wound complications and no sciatic nerve dysfunction. One patient (with pre-operative refractory ischial bursitis) has had a subsequent guided injection as a result of recurrent ischial pain.

An endoscopic technique allows a safe approach to the area of damage in most tears. The benefits of this endoscopic approach, without elevating the gluteus maximus and with the use of endoscopic magnification to protect the sciatic nerve will improve the management of these injuries and reduce the morbidities associated with an open approach. The relative indications for this procedure include a symptomatic complete (at least two tendon) tear of the origin. In general, if the tear is relatively acute, the degree of retraction is not critical as the tissue is mobile and can be brought back to length. In longer standing cases (over 2 months) where there is retraction of greater than 5 cm, it may be difficult to perform the procedure. Any partial tear with chronic bursitis is a candidate for the endoscopic approach, in the author’s opinion.

## SURGICAL TECHNIQUE

**Fig. 4.**
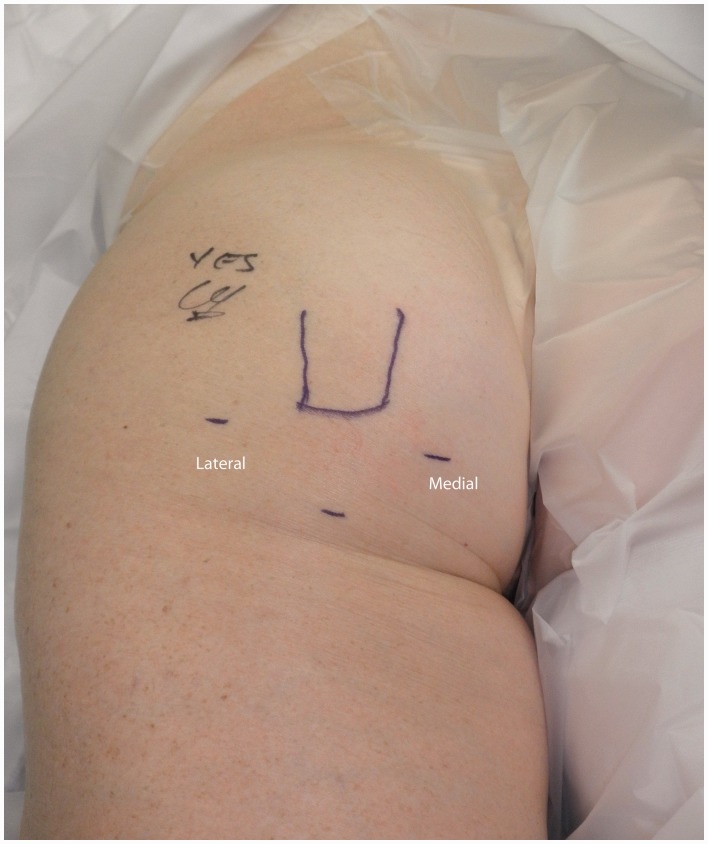
Positioning of the patient in the prone position with the leg draped free. This is of a left hip, positioned prone.

The patient is placed in the prone position after induction of anesthesia, with all prominences and neurovascular structures protected. The posterior aspect of the hip is then sterilized assuring that the leg and thigh are free so that the leg and hip can be re-positioned (Fig. 4).

**Fig. 5.**
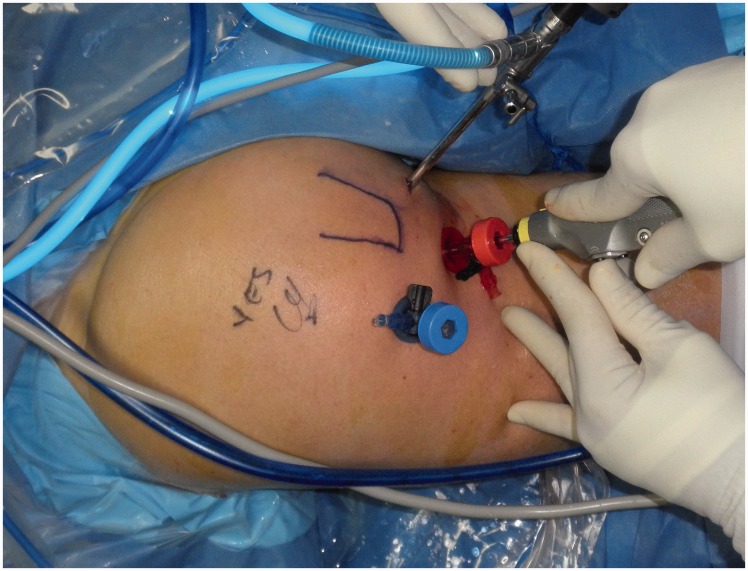
Portals for endoscopic approach with the arthroscope in the medial portal. The shaver is in the distal portal.

**Fig. 6.**
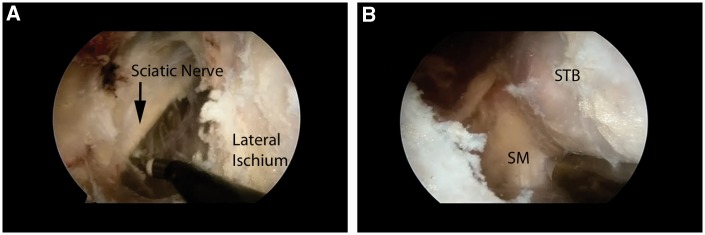
Normal arthroscopic anatomy exposure in a left hip, viewed from the lateral portal. (A) Sciatic Nerve and lateral ischium. Note the tool entering from the medial portal. (B) Inferior ischium depicting the separate attachments of the semimembranosus (SM) and the common biceps and semitendinosus (BST).

Two portals are then created, 2 cm medial and lateral to the palpable ischial tuberosity (Fig. 5). The lateral portal is established first, using blunt dissection with a switching stick, as the gluteus maximus muscle is penetrated and the sub-muscular plane is developed. The prominence of the ischial tuberosity is identified and the medial and lateral borders are located. The medial portal is then established, taking care to palpate the medial aspect of the ischium. A 30° arthroscope is then inserted in the lateral portal and an electro-cautery device is placed in the medial portal. Any remaining fibrous attachments between the ischium and the gluteus muscle are then released, staying along the central and medial portions of the ischium to avoid any damage to the sciatic nerve. The tip of the ischium and the medial aspect are delineated, the lateral aspect is then exposed with the use of a switching stick as a soft tissue dissector. With the lateral aspect identified, the dissection continues anteriorly and laterally toward the sciatic nerve (Fig. 6). Very careful and methodical release of any soft tissue bands is then undertaken in a proximal to distal direction in order to mobilize the nerve and protect it throughout the exposure and ultimate repair of the hamstring tendon.

**Fig. 7.**
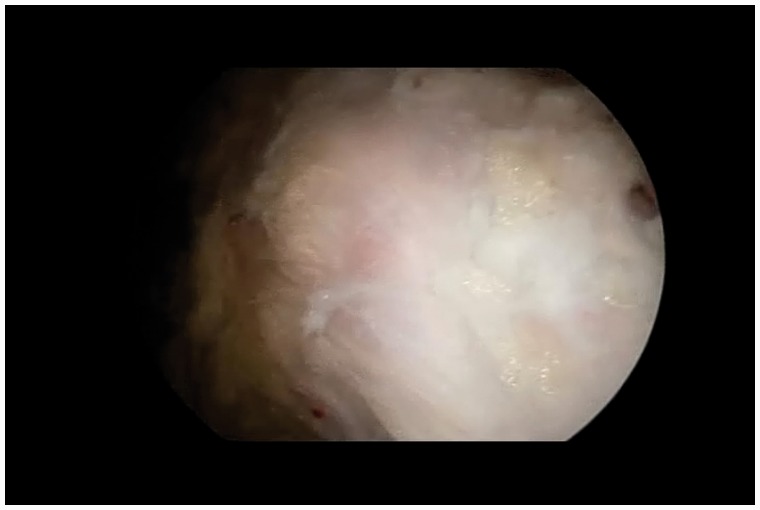
The distal end of the ischium cleared of soft tissue. View is from the lateral portal and shows all of the soft tissue cleared from the hamstring sheath.

With the nerve identified and protected, the tip of the ischium is identified. The tendinous origin is then inspected to identify any obvious tearing (Fig. 7). In acute tears, the area is obvious and the tendon is often retracted distally. In these cases, there is occasionally a large hematoma that requires evacuation. It is especially important to protect the sciatic nerve during this portion of the procedure, as it may be obscured by the hematoma.

**Fig. 8.**
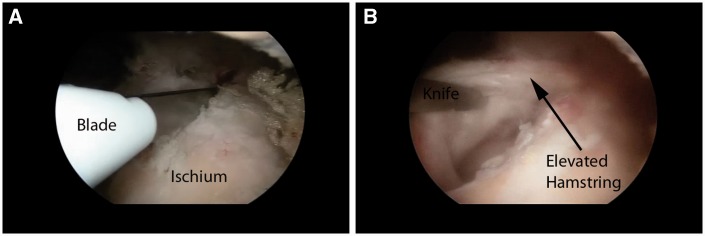
Incision of tendon to explore area of tearing and ischial bursa. (A) Initial knife incision at the most proximal end of the ischium. (B) Exposed detached lateral hamstring complex.

**Fig. 9.**
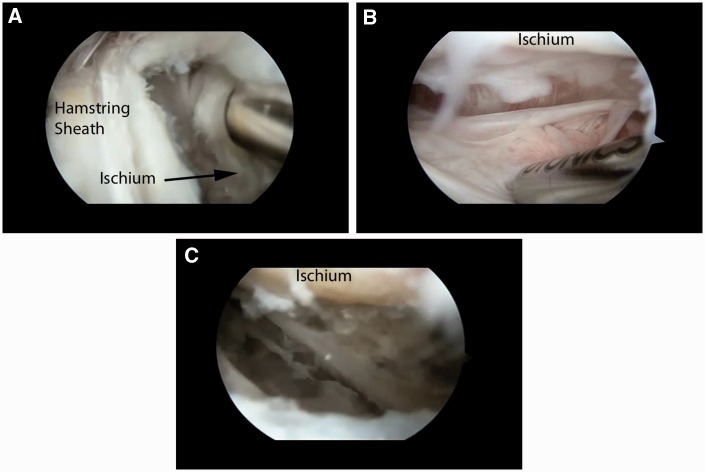
Debridement and exposure of lateral and inferior ischium, including the ischial bursa. (A) Lateral ischium debridement and preparation. Note the tool is serving to retract the detached tissue. (B) Ischial bursa prior to debridement. Note the hypertrophic villonodular tissue surrounding the shaver. (C). Final debridement of bursa. Note the exposed bony surface at the top of the image and the lack of villonodular tissue.

Once the area of pathology is identified (in incomplete tears), an endoscopic knife can be employed to longitudinally split the tendon along its fibers (Fig. 8). This area can be identified through palpation, as there is typically softening over the detachment, making the tissue ballotable against the ischium. The hamstring is then undermined and the partial tearing and lateral ischial wall are debrided with an oscillating shaver. The devitalized tissue is removed and a bleeding corticocancellous bed is prepared in anticipation of the tendon repair. The more distal and inferior ischium and the ischial bursa can also be resected and cleared of inflamed tissues as the lateral ischial tissue is mobilized. By retracting the anterior tissues, the bursa can be entered and debrided (Fig. 9).

**Fig. 10.**
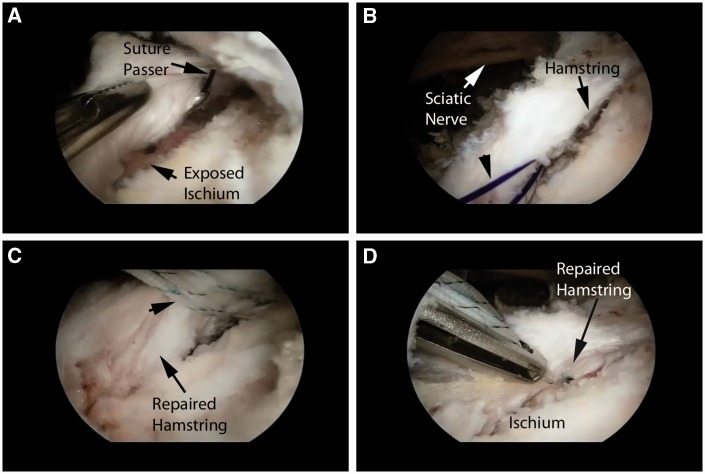
Repair of tendinous avulsion. (A) Prepared surface with suture passer in place. (B) Shuttle suture in place (arrow). Note the proximity of the sciatic nerve to the repair. (C) Final mattress sutures in place in the substance of the tendon. (D) Final tendon repair.

A third portal is then created approximately 4 cm distal to the tip of the ischium and equidistant from the medial and lateral portals (Fig. 5). This portal is used for insertion of suture anchors, as well as suture management. Any variety of suture passing devices can then be used for the repair. The principles are essentially the same as those used in arthroscopic rotator cuff repair. Once all of the sutures are passed through the tissue of the avulsed hamstring, the sutures are tied and a solid repair of the tendon is completed. In general, one suture anchor is used per centimeter of detachment (Fig. 10).

Post-operatively, the patient is fitted with a hinged knee brace that is fixed at 90° of flexion for 4 weeks to maintain the patient non-weight-bearing. The brace will also serve to restrict excursion of the hamstring tendons and protect the repair. At 4 weeks, the knee is gradually extended by about 30° per week to allow full weight bearing by 6–8 weeks, while maintaining the use of crutches. Physical therapy is instituted at this point, with the initial phases focused on hip and knee range of motion. Hamstring strengthening is begun at 10–12 weeks, predicated on full range of motion and a painless gait pattern. Full, unrestricted activity is allowed at approximately 4 months.

## SUMMARY

Historically, the surgical approach to hamstring repairs has not received much attention, as this is not a common area for surgical treatment that is encountered throughout orthopedic training. Those patients with partial tears and chronic bursitis are an even smaller percentage of hamstring problems, with few clinical studies available [22]. With the advent of hip arthroscopy, further development of techniques has allowed us to explore the use of the arthroscope in many previously uncharted areas. The treatment of these hamstring injuries is one such area.

One of the most important aspects in the treatment of proximal hamstring ruptures is early recognition and early treatment. Patients with acute repairs have had better outcomes in the literature when compared with chronic repair [13, 14] Delayed complications of non-operative treatment of proximal hamstring ruptures have been described, and these include knee flexion and hip extension weakness, difficulty sitting and hamstring deformity [23]. The author has used this technique successfully on several acute ruptures as well as chronic partial tears.

Surgical repair of proximal hamstring ruptures also has its inherent risks. With open methods, superficial as well as deep wound infections can occur similar to other surgeries, however, the location of the incision can potentially increase this risk due to the proximity of the incision to urination and bowel movements. With the endoscopic technique, this possibility should be substantially lessened. Additionally the three main nervous structures at risk to iatrogenic injury are the posterior femoral cutaneous, inferior gluteal nerve and sciatic nerves [18, 24]. The sciatic nerve is in close proximity to the ischial tuberosity as it runs along the lateral aspect. With the endoscopic technique, the need for retraction is essentially non-existent since the nerve is identified and visualized during the repair, but no retraction is necessary.

A concern unique to the endoscopic approach is fluid extravasation into the pelvis as a result of the fluid used in the distension of the potential space around the hamstring tendon. Every effort should be made to regularly check the abdomen for any evidence of abdominal distension. In positioning the patient, it is important the abdomen not have any pressure on it such that bolsters are placed longitudinally across the iliac crests and the chest. The anesthesia team should be able to palpate the abdomen periodically by reaching under the patient. In addition, the patient’s temperature should be monitored. Any significant decrease in core temperature may be an indication of fluid extravasation into the retroperitoneal space. Likewise, any unusual blood presure decreases that may be due to fluid compression from retroperitoneal extavasation need to be kept in mind. In general, an attempt should be made to maintain the fluid inflow pressures as low as is feasible for good visualizaiton and an attempt should be made to keep track of fluid ingress and egress volumes to assure that extavasation is avoided.

Through the judicious application of this technique, many of the chronic hamstring injuries and some of the acute injuries previously addressed through a more invasive, open method can be effectively addressed. With this improved technique, it is hoped that a further understanding of hamstring injuries and their sequelae can be further developed.

## CONFLICT OF INTEREST STATEMENT

None declared.
